# Les anomalies congénitales oculaires au Centre Hospitalier Universitaire-Campus de Lomé, Togo

**DOI:** 10.11604/pamj.2021.38.79.21757

**Published:** 2021-01-22

**Authors:** Bénédicte Marèbe Diatewa, Nidain Maneh, Aboubakr Sidik Domingo, Koboyo Esso-Issinam Gnansa, Yannick Francis Amah Ayikoue, Komi Patrice Balo

**Affiliations:** 1 Service d´Ophtalmologie, Centre Hospitalier Universitaire, Campus de Lomé, Lomé, Togo,; 2 Université de Lomé, Faculté des Sciences de la Santé, Lomé, Togo

**Keywords:** Anomalies congénitales oculaires, anomalies systémiques, associations des anomalies congénitales, Togo, Congenital ocular anomalies, systemic abnormalities, associations of congenital anomalies, Togo

## Abstract

**Introduction:**

les anomalies congénitales oculaires sont des entités cliniques rares dans le monde. Le but de cette étude est de décrire les aspects épidémiologiques et cliniques des anomalies congénitales oculaires au Centre Hospitalier Universitaire-Campus de Lomé.

**Méthodes:**

une étude rétrospective a été réalisée de janvier 2016 à décembre 2018 (3 ans) dans le service d´ophtalmologie du Centre Hospitalier Universitaire-Campus de Lomé. Elle a concerné les enfants porteurs d´anomalies congénitales oculaires. Les variables d´étude ont été: le sexe; l´âge au moment du diagnostic; le type d´anomalies congénitales oculaires; la latéralité.

**Résultats:**

sur 2621 enfants examinés durant la période d´étude, 103 (3,9%) étaient porteurs d´anomalies congénitales oculaires. Des 103 patients, il y avait 60 (58,2%) garçons et 43 (41,8%) filles. L´âge moyen de diagnostic était de 16 ± 5,2 mois (extrêmes: 1 mois et 5 ans). L´anomalie congénitale oculaire la plus fréquente était la cataracte (53,4 %). Les atteintes unilatérales étaient prépondérantes (56,3%). Les anomalies congénitales oculaires étaient: isolées (82,5%) ; associées aux anomalies systémiques (11,7%); associées entre elles (5,8%).

**Conclusion:**

ces résultats indiquent que les caractéristiques épidémiologiques et cliniques des anomalies congénitales oculaires se rapprochent de celles rapportées dans la littérature. Cependant, dans notre milieu, la fréquence des anomalies congénitales oculaires et l´âge de diagnostic sont élevés. Il est important de diagnostiquer tôt les anomalies congénitales oculaires dans le but d´assurer la prise en charge et de préserver la fonction visuelle.

## Abstract (en)

**Introduction:**

congenital ocular anomalies are rare clinical entities. The purpose of this study is to describe the epidemiological and clinical features of congenital ocular anomalies at the University Hospital Campus in Lomé.

**Methods:**

we conducted a retrospective study at the Department of Ophthalmology of the University Hospital Campus in Lomé, over a 3-year period, from January 2016 to December 2018. It involved children with congenital ocular anomalies. The study variables were: sex; age at diagnosis; type of congenital ocular anomalies; laterality.

**Results:**

out of 2621 children assessed during the study period, 103 (3.9%) had congenital ocular anomalies. Of these, 60 (58.2%) were boys and 43 (41.8%) girls. The average age at diagnosis was 16 ± 5.2 months (ranging from 1 months to 5 years). The most common congenital ocular anomaly was cataract (53.4%). Unilateral alterations were predominant (56.3%). Congenital ocular anomalies were isolated (82.5%); associated with systemic anomalies (11.7%); associated with each other (5.8%).

**Conclusion:**

these results show that the epidemiological and clinical features of congenital ocular anomalies are similar to those reported in the literature. However, in our Hospital, the frequency of congenital ocular anomalies and patients’ age at diagnosis are high. Early diagnosis is essential to ensure adequate management and preserve visual function.

## Introduction

Les différentes étapes de la mise en place des structures oculaires dans la vie embryonnaire peuvent être altérées, aboutissant aux anomalies congénitales oculaires. Celles-ci sont rares, avec une incidence mondiale de 1 à 2 cas pour 10 000 naissances [1]. Elles sont isolées ou associées à des anomalies systémiques et à un retard de développement psychomoteur [2]. La présente étude a pour but de décrire les aspects épidémiologiques et cliniques des anomalies congénitales oculaires au Centre Hospitalier Universitaire-Campus de Lomé (Togo).

## Méthodes

Il s´est agi d´une étude rétrospective. Elle a été réalisée dans le service d´ophtalmologie du Centre Hospitalier Universitaire-Campus de Lomé, de janvier 2016 à décembre 2018 (3 ans). Elle a porté sur les enfants atteints d´anomalies congénitales oculaires. Les variables d´étude ont été: le sexe; l´âge au moment du diagnostic; le type d´anomalies congénitales oculaires; la latéralité. Toutes les informations ont été recueillies à partir des dossiers des patients. Les données ont été saisies et analysées grâce au logiciel Epi-info version 7.0.

## Résultats

**Données épidémiologiques:** sur un total de 2621 enfants reçus dans le service d´ophtalmologie du Centre Hospitalier Universitaire-Campus durant la période d´étude, 103 (3,9%) étaient porteurs d´anomalies congénitales oculaires. Les garçons représentaient 58,2% (n = 60) et les filles 41,8% (n = 43). L´âge moyen au moment du diagnostic était de 16 ± 5,2 mois (extrêmes: 1 mois et 5 ans). Les données relatives aux tranches d´âge des patients sont rassemblées dans le Tableau 1.

**Tableau 1:** répartition des patients en fonction de la tranche d´âge

Tranche d’âge	Patients	
	**Effectif**	**Pourcentage (%)**
0 -12 mois	34	33
> 12 -36 mois	55	53,4
> 36 -60 mois	14	13,6

**Données cliniques:** parmi les anomalies congénitales oculaires retrouvées pendant la période d´étude, la cataracte était prépondérante (53,4%), suivie du glaucome (19,4%) (Tableau 2). Les anomalies congénitales oculaires unilatérales étaient les plus fréquentes (57,3%). Seuls la cataracte et le glaucome présentaient les deux types de latéralité: unilatéralité et bilatéralité. Toutes les autres anomalies congénitales oculaires étaient bilatérales (Tableau 2). Les anomalies congénitales oculaires isolées étaient les plus fréquentes (n = 85; 82,5%). Des associations entre anomalies congénitales oculaires étaient retrouvées (n = 6; 5,8%); il s´agissait de : la cataracte et la microphtalmie (n = 1); la cataracte et la microcornée (n = 3); la sclérocornée et la microcornée (n = 2). En outre, des associations entre anomalies congénitales oculaires et anomalies systémiques étaient retrouvées (n = 12; 11,7%). Elles concernaient: la cataracte et la persistance du canal artériel (n = 4); la cataracte et la communication interventriculaire (n = 3); la cataracte et la polykystose rénale (n = 1); l´anophtalmie et la persistance du canal artériel (n = 1); l´anophtalmie et le rein unique (n = 1); la microphtalmie et la craniosténose (n = 1); le colobome de l´iris et le spina bifida (n = 1).

**Tableau 2:** répartition des anomalies congénitales oculaires selon la latéralité

Types d´anomalies congénitales oculaires	Latéralité	
	Atteinte unilatérale n(%)	Atteinte bilatérale n (%)
Cataracte congénitale	46(44,7)	9(8,7)
Glaucome congénital	13(12,6)	7(6,8)
Microcornée	0(0)	5(4,9)
Sclérocornée	0(0)	5(4,9)
Anophtalmie	0(0)	4(3,9)
Dystrophie cornéenne	0(0)	4(3,9)
Microphtalmie	0(0)	4(3,9)
Colobome de l’iris	0(0)	2(1,9)
Enophtalmie	0(0)	2(1,9)
Ectopie cristallinienne	0(0)	1(0,9)
Hypoplasie du nerf optique	0(0)	1(0,9)
**Total**	59(57,3)	44(42,7)

## Discussion

La fréquence des anomalies congénitales oculaires varie d’un pays à l’autre. Celle estimée dans notre étude est inférieure à celles retrouvées dans une étude camerounaise (6,65 %) [3] et nigériane (16,4%) [4]. Cependant, dans les pays plus développés à l’instar de l’Egypte, l’Espagne et les Etats Unis d’Amériques dont New York, la fréquence de ces anomalies est inférieure à 1% (0,04 – 1%) [5-7]. Le diagnostic d’anomalies congénitales dans notre contexte est fait à un âge élevé ; même constat fait dans d ’autres travaux d’Afrique subsaharienne [3, 4, 8-10]. La tranche d´âge de diagnostic la plus représentée dans notre étude est celle de > 12-36 mois (53,4%). Elle est suivie de celle de 0-12 mois (33%). Par contre, dans le travail effectué au Cameroun, un taux élevé de patients était noté pour la tranche d´âge de diagnostic de 0 - 12 mois (72,6%); la tranche d´âge de diagnostic de > 12-36 mois comptait 20,7% de patients [3].

L´âge moyen élevé de diagnostic, observé dans la présente étude, a également été retrouvé dans d´autres travaux [3, 4, 11-13]. Dans les pays développés, le diagnostic se fait en période anténatale et dès les premières semaines de vie [14-16]. Les causes du retard de diagnostic dans les pays d´Afrique subsaharienne ont été rapportées dans la littérature; il s´agit entre autres de: l´ignorance par les parents de la maladie; le recours par les parents aux tradipraticiens avant toute consultation en milieu hospitalier; l´insuffisance de sensibilisation de la population; l´absence d´un examen ophtalmologique systématique à la naissance; l´éloignement du centre de santé; le manque de ressources matérielles et financières [12,13]. Dans bon nombre de travaux, il a été noté un taux de glaucome congénital bilatéral plus élevé (51-93%), comparativement à celui du glaucome congénital unilatéral (7-49%) [8, 12, 13, 17]. L´étude réalisée au Cameroun a révélé des taux identiques (2,5%) de glaucome congénital bilatéral et de glaucome congénital unilatéral, et un taux de cataracte congénitale bilatérale (6,3%) supérieur à celui de la cataracte congénitale unilatérale (4,4%) [3]. Les travaux effectués au Nigéria ont montré des taux identiques, d´une part de cataracte congénitale bilatérale et de cataracte congénitale unilatérale (19,55%), et d´autre part de glaucome congénital bilatéral et de glaucome congénital unilatéral (4,35%) [18]. Toutes ces caractéristiques cliniques diffèrent des nôtres. Les différences observées pourraient s´expliquer par l´existence des facteurs environnementaux, de l´hérédité polygénique et multifactorielle et des causes inconnues [5, 19-22]. La bilatéralité des autres anomalies congénitales oculaires, retrouvée dans la présente étude, serait vraisemblablement causée par les facteurs environnementaux et génétiques [19, 23, 24]. Les données de la littérature révèlent que la cataracte congénitale et le glaucome congénital occupent le plus souvent les trois premiers rangs parmi les anomalies congénitales oculaires [3, 7-11, 18, 25]. Nos résultats corroborent cette caractéristique clinique.

La fréquence d´anophtalmie (3,9%) dans la présente étude est supérieure à celles retrouvées dans les travaux menés au Cameroun (0,6%) [3], en Espagne (2,1%) [6] et au Nigéria (2,9%) [18]. Elle est inférieure à celles rapportées dans d´autres travaux (5-12%) [5, 7, 26]. La fréquence de la microphtalmie dans cette étude (4,9%) se rapproche de celle retrouvée dans les travaux réalisés au Nigéria (4,3%) [18]. Elle est supérieure à celle estimée dans l´étude effectuée en Espagne (2,1%) [6]. Elle est inférieure à celles rapportées par d´autres auteurs (5-23%) [3, 4, 5, 7, 9, 26]. La microcornée (4,9%) est retrouvée dans notre étude à une fréquence supérieure à celle notée au Cameroun (3%) [3]. Elle est inférieure à celles déterminées en République Démocratique du Congo (7%) [9] et au Nigéria (5,8%) [18]. Le colobome de l´iris retrouvé dans cette étude a une fréquence supérieure à celles estimées dans les travaux menés en Espagne (0,5%) [6] et au Nigéria (1,4%) [16]. Elle est inférieure à celles rapportées dans d´autres travaux (2,9-10%) [4, 26]. Les études réalisées en Espagne et en République Démocratique du Congo indiquent l´existence de deux groupes d´anomalies congénitales oculaires: les anomalies congénitales oculaires isolées et les associations entre anomalies congénitales oculaires et anomalies systémiques [6, 9]. Outre ces deux groupes, un troisième groupe a été retrouvé dans l´étude effectuée en Egypte [27] et dans la nôtre; il s´agit des associations entre anomalies congénitales oculaires. Des taux moins élevés d´anomalies congénitales oculaires isolées ont été rapportés dans les études menées en Espagne (21%) [6] et en Egypte (48,3%) [27], comparativement à nos données.

## Conclusion

L´ensemble de ces résultats indique que les caractéristiques épidémiologiques et cliniques des anomalies congénitales oculaires se rapprochent de celles rapportées dans la littérature. Cependant, dans notre milieu, la fréquence des anomalies congénitales oculaires et l´âge de diagnostic sont élevés. Il est important de diagnostiquer tôt les anomalies congénitales oculaires dans le but d´assurer précocement la prise en charge et de préserver la fonction visuelle.

### Etat des connaissances sur le sujet

- Des études réalisées dans des pays développés et en voie de développement ont décrit les aspects épidémiologiques des anomalies congénitales oculaires. Elles révèlent que ces dernières sont des entités pathologiques rares;
- De plus, des techniques de détection précoce des anomalies congénitales oculaires ont été développées dans le but d´assurer tôt la prise en charge.

### Contribution de notre étude à la connaissance

- La présente étude rapporte les premières données épidémiologiques sur les anomalies congénitales oculaires au Togo;
- Le diagnostic de ces anomalies est fait à un âge tardif;
- Notre travail révèle l´existence de trois groupes d´anomalies congénitales oculaires: le groupe 1 est celui des anomalies congénitales oculaires isolées; le groupe 2 représente les associations entre anomalies congénitales oculaires; le groupe 3 est celui des associations entre anomalies congénitales oculaires et malformations systémiques.
